# Exploring barriers to and enablers of adequate healthcare for Indigenous Australian prisoners with cancer: a scoping review drawing on evidence from Australia, Canada and the United States

**DOI:** 10.1186/s40352-016-0036-8

**Published:** 2016-05-03

**Authors:** Jessica Olds, Rachel Reilly, Paul Yerrell, Janet Stajic, Jasmine Micklem, Kim Morey, Alex Brown

**Affiliations:** 1 Wardliparingga, South Australian Health and Medical Research Institute, Nort Terrace, 5000 Adelaide, Australia; 2 Centre for Population Health Research, University of South Australia, Adelaide, Australia

**Keywords:** Cancer, Prisoners, Aboriginal, Healthcare, Access, Barriers

## Abstract

**Background:**

International frameworks supported by national principles in Australia stipulate that prisoners should be provided with health services equivalent to those provided in the general community. However, a number of barriers unique to the prison system may hinder the provision of equitable healthcare for this population. In Australia, Indigenous people carry a greater burden of cancer mortality, which the Cancer Data and Aboriginal Disparities (CanDAD) project is seeking to address. During the course of recruiting participants to the CanDAD study, Indigenous Australian prisoners with cancer emerged as an important, under-researched but difficult to access sub-group.

**Methods:**

This scoping review sought to identify barriers and facilitators of access to adequate and equitable healthcare for Indigenous Australian prisoners with cancer in Australia. This review demonstrated a lack of research and, as such, the scoping review was extended to prisoners with cancer in Australia, New Zealand, the United States and Canada. This approach was taken in order to summarise the existing body of evidence regarding the barriers and facilitators of access to adequate and equitable healthcare for those who are incarcerated and suffering from cancer, and highlight areas that may require further investigation.

**Results:**

Eight studies or commentaries were found to meet the inclusion criteria. This limited set of findings pointed to a range of possible barriers faced by prisoners with cancer, including a tension between the prisons’ concern with security versus the need for timely access to medical care.

**Conclusion:**

Findings identified here offer potential starting points for research and policy development. Further research is needed to better elucidate how barriers to adequate cancer care for prisoners may be identified and overcome, in Australia and internationally. Furthermore, given Indigenous Australians’ over-burden of cancer mortality and over-representation in the prison system, further research is needed to identify whether there are a unique set of barriers for this group.

## Background

In June 2014, an estimated 33,971 Australians were in prison. Indigenous Australians made up 27.42 % of this population, despite representing only 3 % of the general population (Australian Bureau of Statistics 2013, 2014). The relative social disadvantage of the Australian prison population as a whole, compared to the general population, is well documented. This includes disadvantage in areas such as housing, employment, education and health (Alan et al. 2011; Australian Institute of Health and Welfare 2013). Chronic illness is of particular concern for this population, with one third of prisoners identified as having a chronic health condition and 2 % ever being diagnosed with cancer (Australian Institute of Health and Welfare 2013). This suggests that for Indigenous Australians who are incarcerated, these vulnerabilities are compounded; they are vulnerable both as prisoners and as members of an Indigenous population already over-burdened by disadvantage and chronic disease (Australian Institute of Health and Welfare 2015).

In Australia, the age-standardised incidence rate for all cancers combined is slightly lower for Aboriginal and Torres Strait Islander Australians (hereafter referred to as Indigenous Australians) compared to their non-Indigenous counterparts, while the age-standardised mortality rate for all cancers is significantly higher (Australian Institute of Health and Welfare and Cancer Australia 2013). A range of factors likely contribute to this higher mortality. Indigenous Australians are diagnosed with more lethal cancers including lung, head and neck, liver and cervical cancers at higher rates and at younger ages than non-Indigenous Australians (Australian Institute of Health and Welfare and Cancer Australia 2013; Chong and Roder 2010). Indigenous Australians are also more likely to be diagnosed with more advanced cancers, there is a higher prevalence of chronic disease comorbidity and a lower likelihood of Indigenous Australians being offered, choosing and completing treatment (Condon et al. 2013, 2014; Cunningham et al. 2008; Moore et al. 2014; Roder 2007).

In order to address this unacceptable disparity, the Cancer Data and Aboriginal Disparities (CanDAD), a large collaborative project involving key cancer-related government and non-government project, is developing a comprehensive system for monitoring cancer incidence trends, management and survival in South Australia. Uniquely, the data system will incorporate Indigenous Australian’s experiences with cancer services to guide continuous service improvement, community engagement, advocacy and research, and provide data infrastructure for health services and population research. During the course of recruiting participants to this study, Indigenous Australian prisoners with cancer emerged as an important, under-researched but difficult to access sub-group. In light of the overrepresentation of Indigenous people in Australian prisons and lack of research regarding prison quality for Indigenous Australians (Rynne and Cassematis 2015), the Aboriginal community governance committee guiding the research argued strongly that this vulnerable group should be included in the range of patient pathways being explored in the project. As such, the aim of the current scoping review is to provide an overview of the existing body of research is relation to Indigenous Australian’s experience of cancer treatment in a prison setting.

For many offenders, prison presents an opportunity to access health services that may not be accessed within the wider community (Butler et al. 2008). Although prisoners in Australia lose access to Medicare and the Pharmaceutical Benefits Scheme, international frameworks from the United Nations (United Nations 1990) and World Health Organisation (World Health Organisation 2007), supported by national principles in Australia, stipulate that prisoners should be provided with health services equal to those provided in the general community (Australian Institute of Health and Welfare 2014). However, a number of barriers unique to the prison system may hinder the provision of equitable healthcare to a population with elevated levels of need (Australian Institute of Health and Welfare 2013). For example, continuity of care can be difficult to achieve given the sudden nature of decisions regarding prison transfers and release. These unique issues have implications for the ability to provide appropriate ongoing care for prisoners, particularly for those with chronic illnesses (Australian Institute of Health and Welfare 2013; Krieg 2006).

With a view to better understanding the patient pathway for Indigenous Australians in prison who have cancer, a review of the relevant evidence on experiences of cancer care, as well as barriers and facilitators of access to care, was carried out. An extensive initial search of peer-reviewed and grey literature limited to Indigenous Australian prisoners with experience of cancer yielded no results, so a more expansive review of evidence relating to the experiences of all prisoners with cancer in Australia, New Zealand, the United States and Canada was carried out. These countries were considered in addition to Australia because similar persistent patterns of health inequities between Indigenous and non-Indigenous people are evident in these countries (Pulver et al. 2010).

The CanDAD project was approved by the Aboriginal Health Research Ethics Committee (04-13-506); the SA Health Human Research Ethics Committee (HREC/13/SAH/55) and the University of South Australia Human Research Ethics Committee (31699).

## Methods

A scoping review was deemed appropriate as a first step in our exploration and understanding of this area, as it provides an indication of the depth and breadth of evidence relating to key concepts and identifies gaps in the literature, in a broad but time limited fashion. While systematic review methods were initially developed for the purpose of synthesising randomised controlled trials, a growing interest in such reviews in the social sciences has led to the development of methods for synthesising a range of evidence types, including qualitative evidence, commentaries and expert opinion (Joanna Briggs Institute 2014; Oliver 2015). Scoping reviews can also incorporate a range of evidence types, but differ from a systematic review in that the evidence is summarised to provide an overview, or map, of the available evidence, rather than a set of synthesised findings (Arksey and O’Malley 2005).

This scoping review followed the steps outlined by Arksey and O’Malley (2005) and further elaborated by Levac et al. (2010). These are: defining the research question; identifying relevant studies; selecting studies; data charting; collating, summarising and reporting results; and consulting with stakeholders. Consultation with stakeholders occurred as a parallel process that informed multiple aspects of the review, including search strategy and interpretation of results.

### Research question

The questions addressed by the review were: ‘What do the experiences of male and female prisoners with cancer tell us about the adequacy of care for prisoners with cancer in Australia, New Zealand, the United States and Canada?’ and ‘What does the available evidence reveal about gaps in care, and barriers and facilitators of access to healthcare for prisoners with cancer in Australia, New Zealand, the United States and Canada?’

### Identifying relevant studies

Data sources included published documents sourced from the websites of government departments and relevant organisations (grey literature), peer-reviewed literature from electronic databases, and consultation with stakeholders including healthcare providers from within and outside the prison system in South Australia.

#### Grey literature

Relevant organisations’ (Table 1) websites were accessed and searched for research reports or other relevant studies or opinion pieces relating to cancer and prison (Table 1).

**Table 1:** List of organisations included in website search

Corrective Services in Australia	Other sites	International
Corrective Services NSW	Australian Indigenous Health InfoNet	Department of Justice (USA)
ACT Corrective Services	Australian Institute of Criminology	Correctional Services Canada
Corrections SA	Australian Institute of Health and Welfare	Department of Corrections New Zealand
Department of Corrective Services, WA	Royal Australian College of General Practitioners	American Cancer Society
Department of Correctional Services, NT	Cancer Council in all states	Canadian Cancer Society
Department of Justice and Regulation, Victoria		Cancer Society of New Zealand

#### Inclusion criteria

Inclusion criteria were broad and included any studies, reports or commentaries relating to the experiences or barriers and facilitators of healthcare for people in prison who have been diagnosed with cancer of any type. Prevalence studies or studies reporting on care for other or unspecified disease groups were excluded.

#### Search strategy

The search was limited to studies published in English between 1990 and 2015 on the basis that more recent evidence is likely to be most relevant to the contemporary policy environment. PubMed, CINAHL, PsychINFO and Scopus were searched for relevant articles. Search terms were broad and included multiple terms for cancers and prisoners (see Table 2). Search terms were modified to correspond with the mesh terms (MH) and thesaurus of each database. Terms were entered as text words in order to achieve a broad, rather than specific, representation of the literature. Limiting the search by address or country of origin did not prove useful, therefore these search terms were omitted.

**Table 2:** Search strategy for Pubmed database

Search	Query
#1 Disease	neoplasms [mh] OR tumor [tw] OR tumour[tw] OR neoplasia [tw] OR cancer [tw] OR oncolog*[tw]
#2 Setting	prisoners [mh] OR prisons [mh] OR criminals[mh] OR prison*[tw] OR inmate [tw] OR jail[tw] OR gaol [tw]
#3	#1 AND #2
	Limitations: English & 1990–2015

### Selection of studies

All search results were entered into Endnote (Thomson-Reuters, 1988–2012). Following the removal of duplicates, titles and abstracts were reviewed by two reviewers (JO, RR), followed by full-text review of those potentially meeting the inclusion criteria. The most common reasons for exclusion were study design (prevalence studies were excluded) and population of interest (studies of prisoners without cancer were excluded).

### Stakeholder consultation

Researchers identified a number of key stakeholders in the prison health and cancer care services who were approached for consultation. These included Aboriginal cancer care co-ordinators at a major metropolitan teaching hospital, a GP with extensive experience working with prisoners and the Nursing Director of state-based prison health service. Stakeholders were asked to identify relevant studies for inclusion in the review (no further studies were identified), and were asked to comment on their experience with access and barriers to care for Indigenous Australian prisoners with cancer, in the context of a broader interview for the CanDAD project. These consultations were used to validate the review findings, and provide an Australian perspective on the international literature.

## Results

**Fig. 1:**
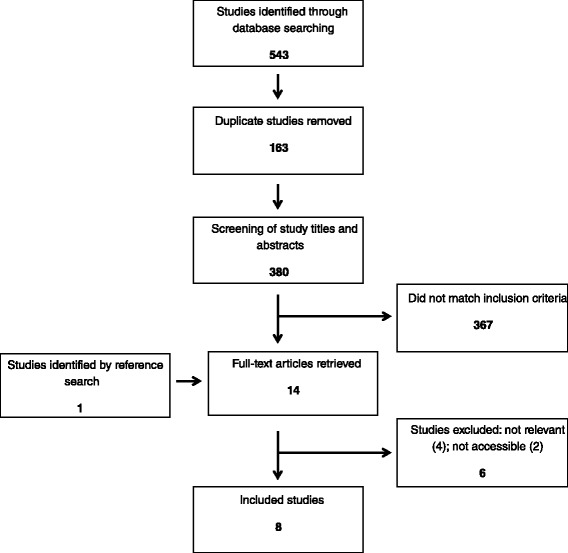
PRISMA diagram detailing results of literature search and study selection

The initial search yielded 543 articles. Duplicates were removed and the titles and abstracts of the remaining 380 articles reviewed. Of these articles, 14 were retrieved for full-text review and eight ultimately met the inclusion criteria (see Fig. 1).

### Data charting

All eight articles identified for data extraction identified issues regarding the access and barriers to care for prisoners with cancer. Of the eight studies, four were commentaries, two case reports, one quantitative survey study and one case–control study. Seven articles were published in the United States and one in New Zealand. Table 3 provides a summary of the articles included in this study.

**Table 3:** Summary of included articles

Study	Study design	Location	Health focus	Issues identified
Courtwright et al. (2008)	Case report	United States	Lung cancer	• Security identified as a barrier to providing equitable healthcare • Healthcare team unable to notify family of patient status • Prisoner unable to engage in activities usually allowed to enhance comfort (television, going outside to smoke, speaking to hospital chaplain) • Cultural conflict between prison & health care system
Lin and Mathew (2005)	Quantitative survey study	United States	Cancer; pain management	• Inadequate pain management • The majority of prisoners with cancer may be under-medicated • Training in cancer pain management as poor or fair by 74 % of Primary Care Practitioners (PCPs) • Common barriers identified by PCPs include concerns about drug misuse/diversion & concerns about patient credibility
Lum (2003)	Commentary	New Zealand	Palliative care (cancer identified as most common diagnosis)	• Treatment often isolating; prisoners may choose to forego • Restricted availability of medical professionals • Prison regulations take precedence over care services (e.g. visitors) • Excessive caution regarding the prescription of medication
Markman (2007)	Commentary	United States	Cancer	• High prevalence of drug abuse made pain management an issue • Unscheduled interruptions in treatment due to sudden transfer or release
Mathew et al. (2005)	Case control study	United States	Cancer	• Pain management problematic due to high prevalence of drug misuse • Terminal illness didn’t guarantee parole
O’Connor (2004)	Case report	United States	Lung cancer; mental health	• Late diagnosis due to prisoner’s complaints not being heard • Prison staff acted in the absence of informed consent from patient • Acting as both representative of the prison and the prisoner presented a dilemma for the service provider
Pillet (2010)	Commentary	United States	Cancer	• Privacy vs. security (guard was always present) • Limited communication between family and practitioners • Family visitation limited • Due to security, practitioners restricted in range of usual activities, including establishing rapport
Wujcik (2010)	Commentary	United States	Cancer	• Goal of oncology team may be in contrast to prison system • Delays in diagnosis & staging compromised outcomes

### Collating, summarising and reporting results

Given the mix of study designs and evidence types, it was not possible to organise the literature according to intervention type or theoretical position. Rather, studies were analysed thematically and coded according to the research questions. That is, data relating to experiences of prisoners reflecting the adequacy of care, gaps in care, barriers and facilitators of adequate care were extracted and presented in narrative form according to dominant themes. Two authors (JO and RR) reviewed the extracted data and agreed upon themes.

### Findings

#### ‘Cultural conflict’ between prison and healthcare systems

Incongruence between the cultures and over-arching goals of the prison and healthcare systems was identified in seven out of eight articles as creating a number of limitations for prisoners’ access to care (Courtwright et al. 2008; Lum 2003; Markman 2007; Mathew et al. 2005; O’Connor 2004; Pillet 2010; Wujcik 2010). This lack of fit was also demonstrated to create barriers to practitioners providing optimal care. The incongruence between systems centred on the regulatory guidelines of the prison, particularly security, versus the delivery of effective, patient-centred health care. As explained by Courtwright et al. (2008):

When the inmate’s condition deteriorates to the point of requiring hospital admission outside the confines of a correctional facility, there is a cultural conflict between the prison system, which embodies punitive and restrictive norms – and the healthcare system – which embraces caring for all the patients’ needs, individual empowerment, and compassion. [p.315]

Several articles identified instances where prison regulations created barriers in terms of delivery and access to client-centred health care (Courtwright et al. 2008; Lum 2003; Pillet 2010; Wujcik 2010). For example, the inclusion of family as a support system is a common aspect of care for cancer patients in the community. This is made difficult in the prison system, where regulations may limit practitioners’ and prisoners’ communication with families. As such, practitioners may be unable to notify family of a prisoner’s health status and prisoners may not be permitted to receive family visits. While compassionate release from prison would address this issue for some prisoners approaching death, both Lum (2003) and O'Connor (2004) report that there is a real risk that this will only occur when it is too late to benefit the patient.

Wujcik (2010) identified cost as a possible barrier to the provision of equitable cancer care:

…the goals of the health management company to contain costs and provide reasonable health services may be in contrast to those of the oncology team who wishes to screen, diagnose and treat cancer at the earliest stage. Delays in diagnosis and staging can compromise the outcome for prisoners.

(Wujcik 2010, p.4)

Inadequate access to timely care and subsequent delays in diagnosis were also identified as a problem by Lum (2003) and O’Connor (2004) due to limited times at which medical professionals were present.

According to Courtwright et al. (2008) and Lum (2003), prison regulations prevent access to interventions and strategies usually employed to enhance patient comfort such as watching television, walking within the hospital to stretch, or going outside for a cigarette. As such, prisoners may find treatment unbearably uncomfortable and isolating, and as a result they may choose to forego treatment. This was reinforced anecdotally during consultations with stakeholders, who recounted examples where prisoners had chosen to postpone or refuse treatment due to the discomfort imposed on them due to being incarcerated, including one prisoner who refused treatment for a life-threatening cancer because he would have to be shackled to the bed in hospital and therefore unable to go outside (Cancer Care Co-ordinator, stakeholder consultation). A similar example was described in O'Connor (2004), where the prisoner explained:

They want me to go to the hospital for pain control. But I am not going. I would rather stay here. Up there they just leave you chained to a bed and forget about you.

[Prisoner ‘W.’, p.69]

O’Connor (2004) highlights that the lack of fit between the prison and healthcare system puts practitioners in a position where they need to constantly advocate for prisoners in order to provide them with appropriate care. One stakeholder who had worked as a general practitioner in prisons, indicated that the need to constantly advocate for prisoners to receive particular treatments was a time-consuming activity and barrier to providing timely, client-centred care that was not encountered in usual practice in the community. O'Connor (2004) also identified that practitioners are often faced with the dilemma of representing both the interests of the prison and the prisoner, making it difficult to provide client-centred care as the interests of the individual and the prison are often in conflict.

The emphasis on security within the prison system appeared to be an overarching issue regarding the lack of fit between prisons and the health care system. All articles identified instances when the emphasis placed on security hindered health care treatment in some way. As well as influencing the issues mentioned above, security regulations created barriers for practitioners to establishing a therapeutic relationship with patients. Due to security concerns practitioners can be limited in their behaviours when treating a patient. For example, Pillet (2010) stated practitioners were not to turn their back on a prisoner, leave anything in the room, or disclose any information about themselves. As such, practitioners are unable to interact with patients in prison in the same way as they might in the general population to establish rapport. Pillet also recognised that prisoners attending medical appointments or receiving treatment are always accompanied by one or more guards.

Stakeholder consultation indicated that this is also a significant issue for healthcare providers in Australia, with one stakeholder stating that, “Security always trumps health” (General Practitioner, stakeholder consultation). However perspectives on the importance of safety, and the possible risk to providers, differed between those working inside the prison, and those working outside the prison or in the general community. While there was acknowledgement by all stakeholders consulted that security concerns were sometimes over-emphasised in patient care, the prison healthcare providers were of the view that doctors who protested against safety measures, such as the restraining of prisoners in hospital, may be naïve to the real risk to safety that some of these prisoners pose (Prison Nursing staff, stakeholder consultation).

#### Drug management

Four articles (Lin and Mathew 2005; Lum 2003; Markman 2007; Mathew et al. 2005) described issues regarding drug management for cancer patients in a prison setting. One of the four articles (Lum 2003) identified issues for the general prescribing of medications such as service providers being apprehensive and excessively cautious about prescribing to offenders. The same article also highlighted that the restricted hours for which a medical professional is present has implications for the management of medications. The remaining three articles identified barriers to pain management for prisoners with cancer. All three of these articles stated that pain management was an issue given the prevalence of drug misuse and abuse in the prison population (Lin and Mathew 2005; Markman 2007; Mathew et al. 2005). Specifically, Markman (2007) draws attention to the dilemma for service providers in distinguishing between genuine pain and drug-seeking behaviours.

Lin and Mathew’s (2005) cross-sectional quantitative survey of prisoners and Primary Care Practitioners (PCPs) focused identifying barriers to cancer pain management in prisons. Prisoners with cancer pain were assessed using the Brief Pain Inventory to establish the level of pain experienced, as well as the Pain Management Index (PMI) to determine whether the strength of prescribed medication was commensurate to the level of pain experienced. PCPs were interviewed using a modified version of the Clinic Staff Survey of Cancer Pain Management to explore the barriers experienced in delivering cancer pain management.

Results demonstrated that 64 % of prisoners with cancer pain had negative PMI scores, indicating inadequate pain treatment. Thirty one percent of PCPs agreed in interviews that the majority of prisoners with cancer pain were under-medicated while 66 % believed the majority received adequate pain treatment, and the remaining 3 % believed the majority were overmedicated. A lack of training was also identified as a barrier to providing cancer pain management to prisoners with 74 % of PCPs rating training in cancer pain management as poor or fair. Additionally, PCPs were asked to identify the top three barriers to providing cancer pain management for prisoners. Most commonly ranked in the top three were concerns about drug misuse or diversion (85 %), concerns about patient credibility (82 %), inadequate assessment of pain and pain relief (68 %), and excessive regulation of prescribing analgesics (60 %). Lin and Mathew (2005) argue it is likely that the barriers identified by PCPs contribute to the under-treatment of pain for prisoners with cancers.

#### Barriers to upholding a patient’s right to informed consent

Four articles (Courtwright et al. 2008; Lum 2003; O’Connor 2004; Wujcik 2010) reported on barriers to prisoners being able to exercise the right to informed consent. O’Connor’s (2004) case report on the psychological therapy of a prisoner with end-stage lung cancer described a situation when the prisoner did not wish to take the recommended stronger form of pain medication as he wished to maintain a clear mind. The prison staff consequently approached the psychologist to persuade the patient to agree to take the medication. On a separate occasion prison staff withheld information that the prisoner had been denied parole and would therefore probably die in prison, precluding him from making fully informed decisions about his own treatment.

Similarly, Courtwright et al. (2008) described a situation where a prisoner with cancer was in a deteriorating condition in hospital. In view of this, contact was made with the prison to seek approval for the prisoner to have visitors. The doctor making the request was advised that approval depended on the prisoner signing a *Do Not Resuscitate* (DNR) and *Do Not Intubate* (DNI) order. This put the prisoner in a compromising position as he wished to consult with his family about the decision to sign a DNR/DNI order but was only permitted visits if he agreed to the order. As such, the prison had placed conditions on the prisoner which diminished his ability and right to provide informed consent.

## Discussion

The constitution of the World Health Organisation enshrines the highest attainable standard of health as a fundamental human right of every human being (World Health Organisation 2006) and the mandate to provide equitable healthcare for prisoners relative to the general community is enshrined in United Nations Declarations (Office of the High Commissioner for Human Rights 1990; United Nations 1990) and the standard guidelines for corrections across multiple jurisdictions (Corrective Services ACT et al. 2012). Quality care is commonly defined as care that is ‘safe, effective, patient-centred, timely, efficient and equitable’ (United States Institute of Medicine 2001). The evidence reviewed here suggests that there are significant barriers in place preventing the achievement of this level of care for people with cancer in correctional facilities.

The initial aim of this scoping review was to determine the state of literature regarding access and barriers to care for Indigenous Australian prisoners with cancer. However, no relevant studies were found. The search was subsequently broadened to include evidence relating to all prisoners with cancer in Australia, New Zealand, the United States and Canada with a view to addressing two review questions: ‘What do the experiences of male and female prisoners with cancer tell us about the adequacy of care for prisoners with cancer in Australia, New Zealand, the United States and Canada?’ and ‘What does the available evidence reveal about gaps in care, and barriers and facilitators of access to healthcare for prisoners with cancer in Australia, New Zealand, the United States and Canada?’

Seven of the eight articles included in the review originated from the United States, possibly reflecting the sheer size of the prison population in that country, considered the largest in the world (National Research Council 2014). Of all the articles included in the review, only O'Connor (2004) and Lin and Mathew (2005) present data on the experiences of prisoners, which may reflect the difficulty in accessing the prison population for research generally. The remaining six articles are commentaries written by health professionals.

The eight articles included in the review highlight a number of key gaps and barriers to access to health care for prisoners with cancer. Perhaps the most salient barrier identified within this review was the prison systems’ tendency to privilege security over the health of prisoners. This appeared to have an overarching effect on impeding access and driving barriers to cancer care for this population. Barriers to accessing high quality healthcare include the presence of guards in appointments, prisoners needing to be shackled to beds (Courtwright et al. 2008), the relocation or release of prisoners without warning (Markman 2007), fewer routine primary health care appointments (Pillet 2010; Wujcik 2010), inadequate training in pain management and concerns about drug misuse preventing the prescription of pain management to cancer patients (Lin and Mathew 2005). The need for corrections to maintain security, and the prisoners’ right to access quality healthcare presents a very real tension that must be managed if prisoners’ right to equitable healthcare is to be realised.

This review also identified instances in which prisoners’ right to informed consent and decision making concerning treatment was compromised (O’Connor 2004). The issue here did not appear to be one of security, but rather an inability on the part of the prison system to adequately understand and take into account the prisoner’s goals and motivations. By definition, being incarcerated involves a loss of freedom, which may be viewed as at odds with the implicit importance of client autonomy in client-centred care (Bauman et al. 2003). However, extending the loss of freedom experienced in prison to the loss of autonomous decision making of a prisoner regarding their own body is inappropriate and at odds with the mandated requirement, and ethical imperative, to provide community-standard healthcare.

While this review focused on the experience of cancer care within prison, research suggests that higher rates of mortality exist for prisoners post-release (Davies et al. 2010 & Spaulding et al. 2011). Possible explanations for this include a lack of access to affordable healthcare beyond the prison setting (Butler et al. 2008) as well as the influence compassionate release may have on the number of deaths that actually occur in custody (Davies et al. 2010 & Spaulding et al. 2011). Although care beyond the prison setting was outside of the scope of this review, the abovementioned research suggests that prisoners’ may face issues with continuity of care upon release from prison. As such, further research is required to determine whether this higher rate can be attributed to compassionate release and/or the barriers prisoners face in accessing and continuing healthcare once released from prison.

## Conclusions

Prisoners are a group with complex needs and high levels of social disadvantage and exclusion. Indigenous Australians are overrepresented in the prison system and experience higher rates of cancer mortality. This review found a very small evidence base and no studies from Australia. Therefore a strong conclusion to be drawn from the limited data is that further rigorous, empirical research is needed to better elucidate how the barriers to adequate cancer care for prisoners may be identified and overcome, in Australia and internationally. In particular, the experiences of Indigenous prisoners with cancer are largely invisible in the research literature. The main themes identified here offer potential starting points for future research and policy development to better align access and service use with best practice for cancer care in Australia.
